# New Insights in Bovine Follicular Cysts

**DOI:** 10.1111/rda.70048

**Published:** 2025-03-08

**Authors:** Alice Carbonari, Nicola Antonio Martino, Matteo Burgio, Vincenzo Cicirelli, Lorenza Frattina, Maria Elena Dell'Aquila, Annalisa Rizzo

**Affiliations:** ^1^ Department of Veterinary Medicine University of Bari Aldo Moro Valenzano Italy; ^2^ Department of Biosciences, Biotechnology & Environment University of Bari Aldo Moro Valenzano Italy

**Keywords:** bovine follicular cysts, GnIH, GPR147, GPR54, Kisspeptin‐10

## Abstract

The aim of the present study was to understand the involved factors in follicular cysts in dairy cows. The study consisted of an in vivo and in vitro approach. The in vivo part, hormonal evaluation (Kisspeptin‐10 [Kp‐10], Gonadotropin inhibiting hormone [GnIH], Luteinizing hormone [LH], Oestrogens [E_2_] and cortisol) was performed in sera of both healthy (H) and cows with follicular cysts (FC). The in vitro part was concentrated on estimating the distribution of GPR54 (Kp‐10 receptor) and GPR147 (GnIH receptor) on cystic and preovulatory follicles. Serum concentrations of Kp‐10, GnIH, LH, E_2_ and cortisol were significantly higher in Group FC compared with Group H. Gene expression analysis showed a reduction in GPR54 mRNA levels in FC compared to preovulatory follicles, while no expression of the GPR147 receptor was detected. The lower presence of GPR54 in FC compared to the preovulatory follicle can be determined by a down‐receptor regulation induced by elevated serum concentrations of Kp‐10 in cows with ovarian FC. Endocrine imbalances of the hypothalamic–pituitary–ovarian axis, characterising FC, may reflect altered patterns of Kp‐10 and GnIH secretion.

## 1 Introduction

Follicular cysts (FC), in dairy cows, are anovulatory follicular structures, with a minimum diameter of 17 mm, persisting for more than 6 days, and interfering with normal ovarian cyclicity (Mutinati et al. 2013; Silvia et al. 2002). The aetiopathogenesis of cystic follicular disease (COD) of dairy cows is currently unclear, although pathological aspects have been long studied. Numerous studies have hypothesized that FC derives from endocrine imbalances of the hypothalamic–pituitary–ovarian axis (Roth et al. 2012; Sciorsci et al. 2003; Van Holder et al. 2006). Significant differences in luteinizing hormone (LH) concentrations and pulse frequency have been identified in cows with ovarian cysts (Van Holder et al. 2006). This imbalance may be correlated with an altered expression of Kisspeptin‐10 (Kp‐10), a hypothalamic neuropeptide involved in gonadotropin secretion (Brown et al. 2012; Jeon et al. 2013; Tena‐Sempere 2006). In fact, Rizzo et al. (2018) revealed that Kisspeptin blood concentrations were significantly higher in cows with FC compared with healthy cows. Researchers have outlined the relationship between stress and the development of bovine FC. During chronic stress (due to management, high milk yielding, over‐nutrition, etc.), activation of the sympathetic system occurs, which causes chronic exposure to low concentrations of catecholamines, which in turn affects reproduction (Rizzo et al. 2011). Moreover, alterations in thyroid activity (Mutinati et al. 2013), vascular dysfunction (Rizzo et al. 2009b), an imbalance between reactive oxygen species and antioxidants (Rizzo et al. 2009a), changes in the expression of heat shock proteins in the granulosa and theca cells of the follicular wall (Velázquez et al. 2011) and altered levels of insulin and/or insulin growth factors (Braw‐Tal et al. 2009) are factors contributing to the onset of cystic ovarian follicles in dairy cows. Gonadotropin inhibiting hormone (GnIH) was discovered to play important roles in reproduction (Tsutsui et al. 2000). This neuropeptide is a hypothalamic dodecapeptide, which acts through the G‐protein‐coupled receptor 147 (GPR147) (Hinuma et al. 2000) and belongs to the RFamide‐related peptide (RFRP) family (Clarke et al. 2008; Kadokawa et al. 2009). The precursor peptide is 173 amino acids in length and is processed into two peptides, RFRP‐1 and RFRP‐3, characterised by arginine and phenylalanine terminals (Clarke et al. 2008; Kadokawa et al. 2009). GnIH has been found in the hypothalamus and pituitary glands, but also in the reproductive organs (uterus, placenta, follicles), liver, spleen, adrenal and parotid glands in female pigs (Li et al. 2012). This peptide inhibits gonadotropin release and is also involved in various pathologies, such as obesity, diabetes (Wahab et al. 2015), pubertal disorders, infertility and polycystic ovarian syndrome (PCOS) (Wang et al. 2018). The aim of the current study is to focus light on some factors involved in FC, based on in vivo and in vitro studies. The in vivo part, hormonal evaluation (Kp‐10, GnIH, LH, Oestrogens [E_2_] and cortisol) was performed in sera of both healthy and FC dairy cows. The in vitro part was concentrated on estimating the distribution of GPR54 and GPR147 receptors on mural granulosa cells isolated from both cystic and preovulatory follicles. The hypothesis of this study is that cows with ovarian FC are characterised by an endocrine imbalance of the hypothalamic–pituitary–ovarian axis, which in turn reflects an alteration of the receptor patterns.

## 2 Materials and Methods

### 2.1 In Vivo Study

All procedures were conducted in accordance with the institutional guidelines on the welfare and use of animals, and with the informed consent of the owner and the ethics committee (protocol No. 30/18). The study was performed on a commercial dairy farm, consisting of Holstein Friesian cows under a semi‐intensive regimen, at 80 ± 15 days postpartum and between the third and fourth lactation (27 kg/day) (mean body weight 550 kg). All cows included underwent routine clinical examination and they were free from parasitic and infectious diseases. Twenty cows diagnosed with FC (Group FC) and 20 healthy cows on heat (Group H) were enrolled in the study. The cows were diagnosed with FC by two clinical examinations, at an interval of 10 days, using transrectal palpation and ultrasonography (SonoSite, MicroMaxx Bothell, WA, USA, linear probe 5–10 MHz, set at 7.5 MHz). Requirements were that the mean follicular cyst diameter was at least 17 mm, wall thickness was < 3 mm, blood progesterone (ELISA kit‐Enzo Life Sciences, Switzerland) was < 1 ng/mL, and that they persisted for > 6 days (Mutinati et al. 2013). Based on these requirements, cows enrolled at the first clinical examination were re‐examined after 10 days and the diagnosis of cysts was confirmed if at that exam, they still met the enrollment requirements. Oestrus was suspected by the presence of oedema of the vulva, clear mucosal vaginal discharge and standing to be mounted. Oestrus was confirmed by the detection of a preovulatory follicle (turgid uterine wall and floating echoic spots) on transrectal palpation and ultrasonography. Blood samples were taken from the coccygeal vein and stored in cold vacutainer serum tubes, at the following times:

- At the second examination in which ovarian FC were confirmed (Group FC).
- On the day of oestrus (Group H).

Blood samples were centrifuged at 1620 *g* for 10 min at 4°C for serum separation. Serum samples were stored in 1.5 mL Eppendorf tubes at −80°C until further analyses. Serum concentrations of GnIH, Kp‐10, LH, E_2_ and cortisol were measured. GnIH concentrations were determined following the manufacturer's instructions, using a bovine‐specific ELISA kit (MyBioSource Inc. San Diego, CA, USA; intraassay/intraassay precision CV% < 10, sensitivity 1.0 pg/mL; no significant cross‐reactivity with GnIH analogues was observed). Kp‐10 concentrations were determined using an RIA kit (Phoenix Pharmaceuticals Inc. Burlingame, CA, USA; range 10–1280 pg/mL; specificity 100%), as previously described (Rizzo et al. 2018, 2019). LH concentrations were quantified by a bovine‐specific ELISA sandwich test (LH DETECT, INRA centre de Tours, Nouzilly, France) and characterised by an intra/intraassay coefficient of variation of 4.1% (*n* = 4) and 8.1% (*n* = 3) respectively, and a sensitivity of 0.06 ng/mL. An oestradiol 17β high sensitivity ELISA kit (sensitivity 3 pg/mL, specificity 100%) (Thermo Fisher Scientific—USA) was used for measuring E_2_ concentrations. Cortisol concentrations were determined using a competitive inhibition enzyme immunoassay with a bovine‐specific kit (CSB‐E13064B Cusabio; detection range 0.049 ng/mL‐200 ng/mL; sensitivity < 0.049 ng/mL; excellent specificity for detection of bovine cortisol; no significant cross‐reactivity or interference between bovine cortisol and analogues; intraassay precision CV% < 8, intraassay precision CV% < 10).

### 2.2 In Vitro Study

Organ collection was carried out at the local abattoir from cows other than those in the in vivo study. In collaboration with the abattoir staff and the veterinary inspector of the facility, 10 ovaries with or without FC were collected after pre‐ and post‐mortem examinations. Any follicular structure with a diameter of at least 2.5 cm in the absence of a corpus luteum was defined as a follicular cyst (Polat et al. 2015), while follicles were classified as preovulatory when they had a diameter of 15 to 20 mm, in the presence of the CL less than 10 mm and uterine tissues were highly turgid, contractile and included transparent cervical fluid (Polat et al. 2015). In order to have a positive control of the expression of the tested genes, five bovine brains were extracted, and the hypothalamus was dissected using the origin of the optic chiasma and the caudal part of the mammillary body as rostral and caudal borders respectively (Graïc et al. 2018). All collected organs were transported in special hermetically sealed and temperature‐controlled transport containers for biological material; transport lasted about 20 min. Upon arrival at the laboratory, total RNA was extracted with the RNeasy Plus Mini Kit (Qiagen, Hilden, Germany) from hypothalamic tissue and mural granulosa cells collected from preovulatory follicles (*n* = 5) and FC (*n* = 5). In detail, RNA was treated with DNase I to exclude any potential genomic DNA contamination. The isolated RNA was used for reverse transcription–polymerase chain reaction (RT‐PCR). The High‐Capacity Complementary DNA (cDNA) Reverse Transcription kit (Life Technologies) was used to convert RNA to cDNA. Each RNA sample (1 μg) was added to 2 μL 10× RT buffer, 0.8 μL 25× dNTP mix, 2 μL RT random primers, 1 μL M‐MLV RT, 1 μL RNase inhibitor and nuclease‐free H_2_O for a total volume of 20 μL and then mixed gently and centrifuged briefly. Reaction tubes were incubated at 10°C for 10 min, then at 37°C for 120 min and finally at 85°C for 5 min. The relative quantification of the transcripts was carried out by Real‐Time RT‐PCR with the StepOne instrument (Applied Biosystems, Foste City, CA, USA). Specific bovine cDNAs were amplified by PCR using the primers shown in Table 1. Real‐Time PCR was performed in a 20 μL reaction volume containing: 10 μL PowerUp SYBR PCR Master Mix (Applied Biosystems, 2×), 200 nM of each primer, 2 μL of diluted cDNA (1:10) and nuclease‐free water up to 20 μL. Cycling parameters were: 95°C for 2 min, 40 cycles of denaturation at 94°C (45 s), annealing at 60°C (45 s) and extension at 72°C (45 s), final extension at 72°C for 5 min. The analysis was carried out in triplicate. Data were collected by using the StepOne Software (Applied Biosystem) and relative quantification was performed using a comparative method after determining the Ct (threshold cycle) values for the reference endogenous control (beta actin) and the target gene in each sample set, according to the 2^−ΔΔ*Ct*^ method. Changes in mRNA expression levels were calculated after normalisation to actin beta. The program calculates the Δ*Ct* and the ΔΔ*Ct* with the formulas below: Δ*Ct* = *Ct*_Mean (beta actin) − *Ct*_Mean (target gene); ΔΔCt = Δ*Ct* − Δ*Ct*_Mean, so that the gene expression level = 2^−ΔΔCt^. Changes in gene expression were reported as percentage changes relative to controls.

**TABLE 1:** Primer pair sequences used for Real‐Time PCR.

Gene symbol	Full name	Accession number	Primer (5′–3′)	Ta (°C)	Length (bp)
ACTB	Actin beta	BT030480.1	CTTCCTGGGCATGGAATCCT	62.1	149
			TTGATCTTCATGTGCTGGGTG	62.3	
GPR54	G‐protein‐coupled receptor 54 (Kp‐10 receptor)	XM_003586292.6	TGGCAATGCAGCCCTTGT	63.0	189
			GAAAAGCGGCACCAACCAG	62.9	
GPR147	G‐protein‐coupled receptor 147 (GnIH receptor)	XM_024986819.2	GTGGCCATTGCTGTGGAAAG	62.8	95
			CCAGATAACCGCGATGGTGA	63.1	

### 2.3 Statistical Analysis

The results are expressed as the mean ± standard deviation (SD). Statistical analyses were carried out using SPSS version 19 (IBM, New York, USA). The Shapiro–Wilk test and Bartlett's test were used to assess the normality and homoscedasticity of the distributions of the continuous variables. The Student's *t*‐test was used for intergroup comparisons of independent variables, and the Pearson's test was used for measuring correlations between hormones. A value of *p* < 0.05 was considered significant.

## 3 Results

### 3.1 In Vivo Study

GnIH, Kp‐10, LH, E_2_ and cortisol concentrations were significantly higher in Group FC than in Group H. The Pearson's test showed a positive correlation between E_2_ and Kp‐10 (*p* < 0.05) in both Group FC (*r* = 0.76) and Group H (*r* = 0.67). Serum concentrations of GnIH, Kp‐10, LH, E_2_ and cortisol are shown in Table 2.

**TABLE 2:** Serum gonadotropin inhibiting hormone (GnIH), Kisspeptin‐10 (Kp‐10), LH, oestradiol 17β (E_2_) and cortisol concentrations (mean ± SD) in cows with follicular cysts (Group FC) and in healthy cows on heat (Group H).

	Group FC	Group H
GnIH (pg/mL)	3575.02 ± 1506.34 a	1413.93 ± 678.97 b
Kp‐10 (pg/mL)	152.6 ± 28.32 c*	102.68 ± 38.27 d*
LH (ng/mL)	1.85 ± 0.84 c	0.63 ± 0.29 d
Cortisol (ng/mL)	17.49 ± 2.33 c	14.39 ± 1.77 d
E_2_ (pg/mL)	10.96 ± 3.5 c*	5.85 ± 1.84 d*

*Note:* a, b: *p* < 0.001; c, d: *p* < 0.05.

* *p* < 0.05.

### 3.2 In Vitro Study

**FIGURE 1:**
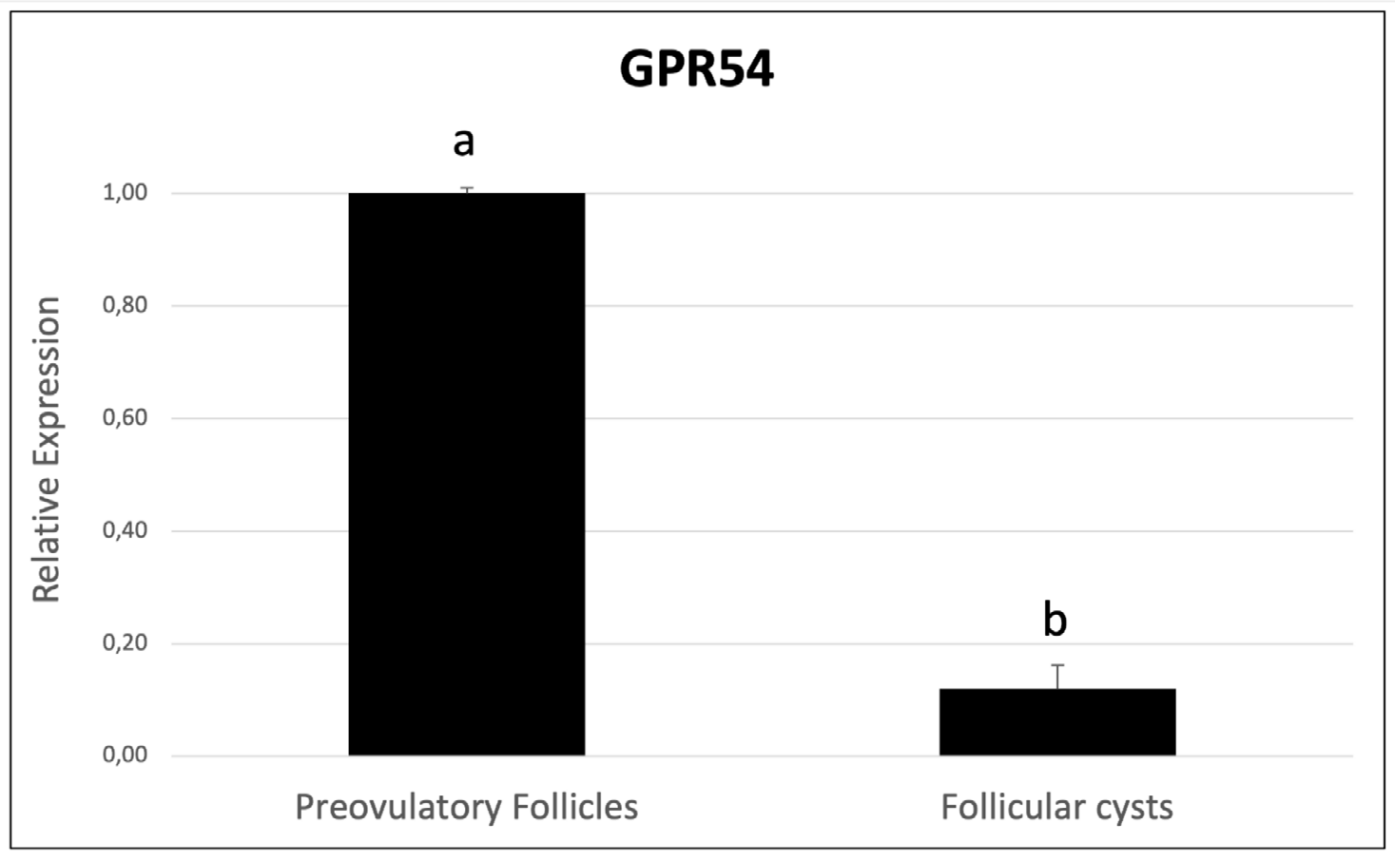
Quantitative real‐time reverse transcription–polymerase chain reaction for GPR54 mRNA relative abundance of mural granulosa cells collected from preovulatory follicles and follicular cysts. The GPR54 mRNA levels were significantly reduced in follicular cysts compared to preovulatory follicles. Bars with different letters indicate statistically significant differences: a, b: *p* < 0.05.

Based on the evident presence of different serum concentrations of Kp‐10 between FC and H cows, the involvement of GPR54 and GPR147 receptors was analysed through the analysis of mRNA levels by quantitative PCR, starting from mural granulosa cells collected from preovulatory follicles and FC. As reported in the Figure 1, the results demonstrate a significant reduction of GPR54 expression of about 8‐fold in FC compared to preovulatory follicles, whereas there was no expression of GPR147 receptor in these samples. Furthermore, in hypothalami, which served as positive controls of the tested transcripts, the expression of both GPR54 and GPR147 receptors was detected.

## 4 Discussion

As far as we know, this is the first study evaluating the concentrations of GnIH and Kp‐10 in both physiological (healthy cows on oestrus) and pathological (cows with FC) conditions of dairy cows. GnIH and Kp‐10 are considered modulators of the hypothalamic–pituitary axis. Kp‐10 stimulates LH release, affecting both GnRH neurons and/or by means of a direct effect on the pituitary gonadotrophs (De Roux et al. 2003; Wang et al. 2018). The present study showed that Kp‐10 serum concentrations were significantly higher in the FC group if compared with the H group. With respect to Kp‐10, the result was consistent with the studies of Jeon et al. (2013) and Rizzo et al. (2018), in which Kp‐10 was measured in women with PCOS and bovine FC, respectively. This is explained by the higher E_2_ concentrations found in the cystic groups, which stimulate Kp‐10. The high concentration of Kp‐10, in turn, stimulates LH release. It is known that cows with cysts have higher serum LH concentrations, with an increase in both frequency and amplitude of LH pulses, compared with cows undergoing ovulation (Garverick 1997; Silvia et al. 2002; Van Holder et al. 2006). Additionally, cysts secrete higher concentrations of oestradiol than follicles undergoing ovulation (Van Holder et al. 2006). The present study supports these results, in that higher concentrations of E_2_ were observed in the FC group than in the H group, and these values were positively correlated with Kp‐10 concentrations. It is conceivable that the higher concentrations of Kp‐10 in the FC group were induced by E_2_ secreted from cysts and Kp‐10 contributed to the maintenance of the cysts because it stimulated LH release. Altered expression of receptors could disturb the endocrine system and prevent ovulation from taking place. The in vivo outcome is also supported by the result obtained in vitro; in fact, both at the level of the preovulatory follicle and the FC, the expression of the GPR54 receptor was found, suggesting that the Kisspeptin signalling may also act directly at the ovarian level. The result obtained in the current study regarding the presence of GPR54 receptors on ovarian structures came in agreement with those conducted by other research groups in different animal species and conditions such as rat (Castellano et al. 2006); siberian hamster (Shahed and Young 2009); dog (Cielesh et al. 2017); pig (Basini et al. 2018) and cattle (Mattar et al. 2023). Furthermore, the lower presence of GPR54 receptors in the FC could be explained by considering the higher concentrations of Kp10 in cows with FC compared with those found in the serum of healthy cows. It is conceivable that the higher concentrations of Kp10 might induce receptor down‐regulation on the FC. In another study, a reduced mRNA expression of the Kp10 receptors was demonstrated in cumulus cells of patients with PCOS, although no difference was identified in mural granulosa cells (Blasco et al. 2019). To the best of our knowledge, no studies report the presence of this type of receptor in FC in cattle; therefore, the data obtained in the present study could help in understanding the follicular dysfunction. There are currently no reports regarding GnIH serum concentrations, either in humans or animals. Shaaban et al. (2018) reported that the expression of GnIH mRNA in the dorsomedial hypothalamic nucleus, after the induction of PCOS in a rat model of constant light exposure, was lower than that in controls. In this case, PCOS was induced, although the naturally occurring condition might behave differently. In the present study, serum concentrations of GnIH in the FC group were higher than those in the control group. It is plausible that this increase was due to stress. In fact, a close correlation between stress and the development of bovine FC has been shown (Mimoune et al. 2019). This is in accordance with our results, which showed higher cortisol concentrations in the FC group than in the H group. In turn, the stress condition influences the release of GnIH; it was recently shown that stress induces proliferation of GnIH‐immunoreactive cells and increases GnIH gene expression in the hypothalamus (Kirby et al. 2009). Moreover, Gingerich et al. (2009) have demonstrated that corticosterone administration increases GnIH mRNA expression levels in a cell line derived from rat hypothalamus.

Although most of the studies in mammals, birds and fish have shown the inhibitory action of GnIH in the hypothalamic–pituitary–gonadal axis, several studies have shown its stimulatory action (Ducret et al. 2009; Ubuka et al. 2012; Ubuka and Parhar 2018).

GnIH may induce LH release by stimulating the activity of aromatase neurons and increasing neuroestrogen concentration in the hypothalamus, as well as stimulating the electrophysiological activity of GnRH neurons and GnRH release (Ubuka and Parhar 2018).

It is also possible that the stimulatory effect of GnIH on the pituitary is due to the down‐regulation of GPR147, the internalisation of the GPR147 receptor by a high concentration of GnIH, and changes in the number of receptors depending on reproductive and developmental stages and endogenous sex‐steroid levels (Amano et al. 2006; Biran et al. 2014; Di Yorio et al. 2016; Osugi et al. 2012; Shahjahan et al. 2011; Wang et al. 2015). Therefore, high concentrations of E_2_, found in the present study, may downregulate and/or desensitise receptors at GnIH nuclei.

Analysing the results obtained with the research of GPR147 receptors for GnIH, it is noted that their presence in this study was not found in the bovine ovaries of both groups examined. There is no information in the bibliography on the location of this type of receptors at the ovarian level in the bovine species. In a study by Li et al. (2012) it is stated that the presence of these receptors and the hormone can be found instead in pig ovaries.

Therefore, as the literature has already suggested, it is conceivable that in bovine FC there is an altered hormonal and receptor pattern. Stress causes an increase of GnIH, but there is a concomitant increase of Kp‐10, due to the high oestrogen concentrations. This hormonal pattern could increase LH, that in turn stimulates E_2_ release and consequently supports the maintenance of the cyst.

## 5 Conclusions

The results obtained in this study show that Kp‐10 receptors (GPR54) are present in bovine ovaries. The different distribution of GPR54 receptors, greater in the preovulatory follicles and less in the FC ones, suggests that the increased presence of circulating hormone in the course of cystic pathologies induces a phenomenon of receptor down‐regulation in the FC. GPR147 receptors for GnIH are absent in ovarian structures. This, compared to increased serum hormone levels in cows with ovarian FC, suggests that GnIH is not directed to ovarian structures, but it induces an increase in LH release. With this in mind, more studies on the subject will certainly have to be carried out, analysing other aspects of the hormonal interaction with the cells of the hypothalamus–pituitary–gonadal system. In addition, this study opens the door to further studies in which substances with antagonistic activity to Kp or GnIH can be tested for use as therapy to resolve FC.

## Author Contributions

A. Carbonari and N. A. Martino performed the in vivo and in vitro study, wrote and revised this manuscript. M. Burgio and V. Cicirelli contributed to writing, reviewing and editing the manuscript. L. Frattina performed analysis and data curation. M. E. Dell'Aquila and A. Rizzo provided project administration, conceptualisation and supervision. All authors have read and agreed to the published version of the manuscript.

## Conflicts of Interest

The authors declare no conflicts of interest.

## Data Availability

The data that support the findings of this study are available from the corresponding author upon reasonable request.
